# Social seeking declines in young adolescents

**DOI:** 10.1098/rsos.170029

**Published:** 2017-08-09

**Authors:** Indu Dubey, Danielle Ropar, Antonia F de C. Hamilton

**Affiliations:** 1 Institute of Cognitive Neuroscience, University College London, Alexandra House, 17 Queen Square, London WC1N 3AR, UK; 2 School of Psychology, University of Nottingham, University Park, Nottingham NG7 2RD, UK

**Keywords:** social seeking, social motivation, atypical development, choose a movie, young adolescents

## Abstract

The desire to engage with others is an important motivational force throughout our lifespan. It is known that social behaviour and preferences change from childhood to adulthood, but whether this change is linked with any changes in social motivation is not known. We evaluated 255 typically developing participants from ages 4–20 years on a behavioural paradigm ‘Choose a Movie’ (CAM). On every trial, participants had a choice between viewing social or non-social movies presented with different levels of effort (key presses/screen touch required). Hence, participants chose not only the movie they would watch but also how much effort they would make. The difference between the effort levels of the chosen and not chosen stimuli helps in quantifying the motivation to seek it. This task could be used with all the age groups with minimal adaptations, allowing comparison between the groups. Results showed that children (4–8 years), older adolescents (12–16 years) and young adults (17–20 years) made more effort to look at social movies. Counterintuitively, this preference was not seen in young adolescents (around 9–12 years), giving a U-shaped developmental trajectory over the population. We present the first evidence for non-monotonic developmental change in social motivation in typical participants.

## 1. Introduction

Human social interaction depends on both abilities and motivations, including recognition of faces and emotions, understanding of others' thoughts and the desire to engage with other people. Our social behaviour and preferences change through development. Recent work suggests that some of these social ‘abilities’ develop non-linearly over childhood, which has both cognitive and clinical implications.

Performance on face recognition tasks either dips or reaches a plateau around age 12 years ([1,2] but see [3]). Emotion recognition using facial expressions might decline at age 11–12 and stabilize at age 15 [4], while emotion recognition through body movement may reach a plateau at age 8.5 and stay the same through adolescence ([5] but see [6]). Preference for ‘attractive’ facial features increases between ages 4–9 years but dips at 10–14 years before reaching adult levels [7].

While the development of cognitive and information processing has been examined as described above, motivational processes have received less attention. There is now increased interest in quantifying and understanding motivational processes. Social motivation can be subdivided into three components: *Social orientation*—the ability to identify and orient to social cues; *social seeking*—making effort to seek pleasurable social interactions; and *social maintenance*—working to foster social bonds [8]. Of these three, social seeking is the least explored component of social motivation and will be the primary focus of the current paper.

Researchers have attempted to measure social seeking using behavioural methods that primarily compare the reward ‘incentive’ value of social and other competing stimuli. In some of these paradigms, participants chose who to gamble with, other person or computer [9], or chose whether to see a social (smiling faces) or non-social movie (household objects) [10]. These tasks showed that typical adults chose social stimuli over non-social even when it comes at the cost of monetary loss or higher effort. An alternative behavioural paradigm is the social incentive delay (SID) task [11–13]. In this task, participants are cued to the social reward (happy face with varying levels of intensity) which they will receive for a fast keypress. They then have the chance to hit a key on seeing a target, and the speed of response is taken as measure of how much they want the reward. It is reported that participants' reaction time to the happier faces is shorter than to the less happy faces [14]. Several studies have used this measure of social seeking with mixed results. The data from both Flores *et al.* [12] and Kohls *et al.* [11] suggest that incentive conditions, either social (smiling faces for Flores *et al.* and smile and thumbs-up gesture for Kohl's *et al.*) or non-social (money for Flores *et al.*), were more motivating than the non-incentive conditions (no reward for Flores *et al.* or neutral facial expression for Kohl *et al.*). Flores *et al.* [12] also compared the two conditions and found that the reaction time was faster for the social conditions than non-social. On the other hand, Cox *et al*. [13] found that participants' reaction times were fastest for the non-social (candies) than for the social condition (smiling person).

Overall, though current evidence suggests higher reward value for social stimuli in typical adults, none of the above-mentioned behavioural studies explored whether the motivation to seek social stimuli is the same during adolescence or childhood. The beginning of several biological changes makes adolescence a sensitive period in social development [15]. This is a time when the brain undergoes significant reorganization that can result in social sensitivity influencing adolescents' behaviours. A study by Demurie *et al*. [16] explored the performance of children and adolescents between ages 8–16 years on the SID task. They found that the typical children and adolescents gave higher subjective rating for social than non-social incentive on a five point Likert scale measuring how motivating or satisfying the task was. Despite this, the social reward (smiling faces or pictogram) had no greater influence on the reaction time of the participants than the non-social incentives (money), suggesting no greater reward value for social stimuli in this group.

Although there is limited literature focusing solely on typical children and adolescents, there are several studies where typical populations have been used in a comparison group for clinical groups (e.g. autism spectrum disorders—ASD). These studies can help to understand the reward value of social stimuli in typical development. One study including typical adolescent participants (mean age 14 years; range 10–21 years) showed a high reward value was expressed for social stimuli (interacting with others) on a subjective rating scale [17]. However, on the behavioural tasks social/monetary incentive delay and approach-avoidance, either they showed no preference for social or non-social stimuli (money versus faces and images of real person versus cartoons) [18,19], or they showed a higher preference for non-social stimuli (cars versus faces) [20,21]. These findings are different from what is found in typical adults.

There is only one study by Kohls *et al.* [22] evaluating social seeking in typical children (8–12 years) using an incentive based go/no-go task. They found reduced error for both social (faces) and non-social (money) reward conditions compared to no-reward condition, however the improvement was highest for the monetary incentive group compared to the social incentive group. This suggests that, unlike adults (as seen in previously discussed studies), typical children prefer non-social stimuli over social stimuli.

In younger children, it is again helpful to look at the performance of the control group in studies with clinical groups. The data from these studies show mixed results. Deckers *et al.* [23] found that 7–12 year old typical children do not show any preference for social (images of faces) over non-social stimuli (images of landscape), whereas Stavropoulos and Carver [24] found that 6–8 year old typically developing children show higher reward value for social (faces) than non-social (arrow mark) stimuli. Therefore, it is difficult to conclude if social stimuli have higher reward value during early years for typical children or if there might be any developmental changes in social seeking over age. To fill the gap in the literature, the current study measures social seeking in a large sample of more than 250 healthy participants between ages 4–20 years. We believe that understanding typical development of social seeking will provide a point of reference for understanding atypical social motivation.

We can distinguish several possible developmental hypotheses for how social seeking might change over the 4–20 years age range. First, there might be no change with a consistent level of social seeking at all age ranges. However, this is not consistent with the mixed evidence for a social preference in children, summarized above. Second, there might be a linear change with a gradual increase (or decrease) in social preference as children get older. Finally, there might be a non-monotonic change with high social preference in young children and adults contrasting with lower social preference in adolescents or young adolescents. This would be consistent with some of the data on face processing reviewed above which suggests non-monotonic changes in performance. The present paper reports an exploratory study which did not set out to test one of these hypotheses over the others, but rather reports the patterns of results found over a large dataset.

In order to explore social motivation over a wide age range, we need to consider what is the most suitable method for quantifying the motivation to engage with another person. The methods used in the above-mentioned studies have several limitations. For example, the commonly used SID task assumes that the motivation to seek social contacts can be measured in terms of reaction times. However, developmental changes in attention and motor control (which determine reaction time) could make it hard to measure preference with a reaction time task. Also, as suggested by Bolles–Bindra–Toates theory of incentive motivation, ‘seeking’ might be a complex series of psychological events that involves (i) learning the association between source of pleasure and cues, (ii) motor or cognitive readiness to take action and (iii) subjective state of the organism that determines level of effort he might make [25]. This goes beyond simple anticipation of pleasure, as evaluated by SID. Furthermore, anticipation might not always result in the same level of effort to seek the stimulus. Hence, we believe that SID might not be the best measure of social seeking behaviour.

Some of the other tasks as used by Shore & Heerey [9], Silva *et al.* [19] or Ewing *et al.* [20] presented example stimuli while the participant made a decision to seek more of its category. Here it is difficult to know if the behavioural response of the participants was influenced by the low-level features (colour, brightness etc.) of the specific stimuli seen by the participant or the general category of the stimuli i.e. social or non-social category. The Choose-a-Movie (CAM) task as used by Dubey *et al.* [10,26] overcomes these limitations. It focuses on the seeking behaviour (learning the association and making effort to see the stimuli) and does not present stimuli while the decision is made, hence controlling influence of low level features of the stimuli on decision. Most important of all, this tool can easily be used with children as young as four, allowing us to explore development across a range of ages using the same task. Therefore, in the current study we used the CAM task to explore the development of social seeking from early childhood through to young adulthood.

## 2. Methods

### 2.1. Participants

We recruited 255 participants ranging in age from 4–20 years for this study. Data were collected in five different cohorts [27] (see table 1 for details), some of which included comparison groups with high functioning autism. Only data from typical participants are presented here. These participants came from a mixed socio-economic and cultural background. The 4–12 year old participants (cohorts 2 and 5) were recruited though the Summer Scientist week (a public engagement programme) organized by the University of Nottingham. Some participants between ages 10–12 and all the other 12–17 year olds (cohort 3) were recruited by contacting local schools. The participants aged 18–20 years (cohorts 1 and 4) were contacted through posters in the university. General health information such as whether the participant had any clinical condition or behavioural problems was noted from the primary caretaker if the participant was under 18, and participants with clinical or behavioural problems were not tested further. The participants over age 18 self-declared not to have any significant psychiatric/medical condition. Sample size for each individual cohort was determined before data collection began. All available data for typical participants aged 4–20 years old were included in the present paper. No adults who declared themselves to be healthy or children whose parents signed the consent forms and declared them healthy were excluded at any later stage. No data point was excluded on the basis of response pattern or any other characteristic of the participant. The data labelled as cohorts 1 and 3 in the table below have also been reported as part of other samples [10,26]. For the clarity of expression, in the rest of the paper we refer to participants aged 4–8 years as ‘children’, those aged 9–12 years as ‘young adolescents’, those aged 12–16 years as ‘older adolescents’ and those aged 17–20 years as ‘young adults’.

**Table 1.** Participant characteristics.

cohort	CAM and setting	number	female : male	age in years (M, ±s.d.)
cohort 1	CAM 1, lab setting	30	18 : 12	18–20 (18.60, ±0.72)
cohort 2	CAM 2, public engagement event	100	52 : 48	4.05–11.11 (8.61, ±1.69)
cohort 3	CAM 2, quiet room in school	40	4 : 36	11.04–16.02 (13.71, ±1.13)
cohort 4	CAM 2, lab setting	32	20 : 12	18–20 (18.78, ±0.71)
cohort 5	CAM 2, public engagement event	53	28 : 25	4.0–11.03 (5.66, ±0.47)
total		255	122 : 133	4–20

### 2.2. Stimuli

Participants in this study viewed two sets of movie stimuli: social direct gaze movies and object movies. There were 10 movies in each set and all of these movies were three seconds in length. The direct gaze movies showed an adult actor smiling directly toward the camera; the object movies showed common household objects slowly rotating on a turntable. The stimuli were made originally by the authors. For the social stimuli, postgraduate students (age 24–35) took part as actors. These actors were asked to imagine that they were sitting in a café when a friend called them. To facilitate the imagination a friend/partner of the actor was asked to call the actor's name. The actor then responded by looking up towards the camera and smiling.

These stimuli were then rated by 15 undergraduate students for genuineness, friendliness, likeability, attractiveness and naturalness. Ten smile movies (equal numbers of males and females) with the highest average rating on these dimensions were then selected as social stimuli. For the non-social stimuli, pairs of regular household objects (brush–paint, shampoo–loofah, coffeemaker–cup etc.) were placed on a small turntable. These objects were then video recorded while slowly rotating on the turntable. Movement was induced in the object videos to match them to the social videos in which models looked up and smiled. Ten of these object movies were selected as the final non-social stimuli. All the movies—social and non-social—were saved at 320 × 180 pixels' resolution and were trimmed to 3 seconds' duration.

### 2.3. Choose-a-movie task

Participants completed the CAM task on a laptop computer running Matlab and Cogent. There were two slightly different versions of the CAM tasks to suit the attention span and cognitive abilities of the participants. Version 1 was completed only by the participants in cohort 1. The simpler version 2 was completed by all the other cohorts. Here we describe the basic task structure which is similar across both versions, and then detail the small changes between the two versions.

The CAM task always starts with association trials. On each of these trials, participants saw a coloured box on the left or right of the screen with one, two or three locks on the box. He/she opened the locks by pressing a key (version 1) or touching it (version 2) and saw a linked movie. Through these trials, they learnt that there was a consistent mapping between the colourful boxes and the categories of movie ‘hidden inside’ each box. For example, a green stripy box always contained movies of smiling people and the pink spotty box always contained movies of rotating objects. The mappings between boxes and stimuli were counterbalanced between the participants. Through the association trials participants also learnt that removing one lock required either one key-hit (version 1) or one finger touch to the screen (version 2). Thus, removing three locks needs three distinct actions which is relatively more effort than removing one lock with one action.

**Figure 1.**
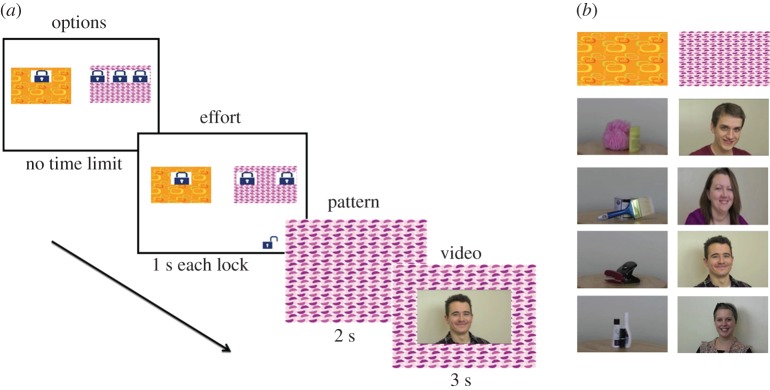
Task and stimuli. (*a*) On each trial, participants see two coloured boxes with one to three locks on each box. They can choose which locks to open (effort stage) by pressing a key or touching the screen. When all locks are removed from a box, the participant can see one of the movies linked with that box. (*b*) The two different patterned boxes were linked to two categories of stimuli—objects and social. Four of the 10 exemplars within each category are illustrated.

Participants then completed the choice trials, in which they saw two coloured boxes on the screen with one, two or three locks on each box (figure 1*a*). They could choose to open one of the boxes to see the movie associated with it. Box location and number of locks were fully counterbalanced as detailed below. On each trial, a participant could choose to open the box with fewer locks (requiring less effort) or the box with more locks (requiring more effort). Thus, participants could make a trade-off between the effort required to open the box and their preference for a movie category.

Two slightly different versions of the task were used because version 1 was developed for use with adults, and was later simplified for use with children. The differences between the two versions of the task were as follows. (i) In version 1, participants completed six additional ‘practice trials’ with a choice of two boxes, before the main choice trials. These trials were not analysed and so were dropped from version 2. (ii) In version 2 of the CAM task, an additional step of instructions was added to ensure that participants clearly knew that each box was linked with one set of movies. In this instruction trial participants were shown a box on the left side of the screen and were asked to press a key to see six images from the associate movie (e.g. green box was shown on left and when key was hit six images taken from the original stimuli video appeared on the right side of the screen). This step was repeated for both the stimuli boxes. (iii) In version 1, participants responded with a key-hit but in version 2 they responded by touching the item on a touch screen. (iv) In version 1 participants completed 180 experimental choice trials; 60 trials gave a choice between social (direct gaze) movies and object movies, and these trials are analysed here because they closely match version 2 of the task. The remaining 120 trials that look at the choice between social averted gaze versus object and social averted gaze versus direct gaze, are reported elsewhere [10]. In version 2, participants only saw the choice between social movies of a smiling adult making direct gaze and movies of objects (60 trials). (vi) In both versions, the video stimuli were the same and the number and location of the locks and boxes were fully balanced. The details of the differences between the two versions are given in table 2.

**Table 2.** Comparison of CAM versions 1 and 2.

variable	CAM 1	CAM 2
contrasts	social direct gaze versus object	social direct gaze versus object
	social averted gaze versus object	
	social direct gaze versus averted gaze	
trials	60 for each contrast	60
stimuli	smiling adult faces and household objects	smiling adult faces and household objects
effort trial distribution (lock difference from right to left for set of 60 trials)	−2 (16 trials)	−2 (12 trials)
	−1 (8 trials)	−1 (12 trials)
	0 (12 trials)	0 (12 trials)
	1 (8 trials)	1 (12 trials)
	2 (16 trials)	2 (12 trials)
response	key press (Z for box on left and M for box on right)	screen touch (touch the box on left or right)
association trials (trials showing only 1 stimulus on screen to open)	5 each stimulus	5 each stimulus
familiarization trials (practice choice trials)	6	none
instructions about each box having 1 stimulus category	no	yes
duration	45–50 min	18–20 min
trial progress indicator	none	a bar at the bottom suggesting how much of the task is over
breaks	1 break screen at the middle of the task	3 break screens suggesting to participant that they were doing well

### 2.4. Procedure

Participants in cohorts 1 and 4 were tested in a quiet room on a university campus. Children in cohort 3 were tested in a quiet room in their own school. Cohorts 1, 4 and 3 received an appropriate inconvenience allowance after the study was complete. Children in cohorts 2 and 5 were tested in a quiet space as part of a children's Summer Scientists week event and received small goody bags at the end of the session. Participants (cohorts 1, 3 and 4) were provided with verbal instructions and then completed the association trials. They were then able to continue with the choice trials at their own pace without further assistance. Child participants (cohorts 2 and 5) were verbally instructed as to what they needed to do and completed the association trials in the same way. The experimenter remained at the same desk as the child throughout, to encourage the child to complete the choice trials.

## 3. Data analyses

The data collected over 60 choice trials from each participant include an individual's age and gender, together with the choice they made on each trial. Two age-related predictors were calculated: zAge—zero-meaned participant age in years, which is the raw ages with the sample mean subtracted so that the whole population has a mean age of zero; zAge^2^—the same value squared. These allow us to construct linear and quadratic models of how age relates to social seeking.

Our primary analysis is a *logistic regression*, where we constructed a mixed-level logistic regression model including all trials and all participants. We tested how the choice to open (or ignore) the box on the left for each trial could be predicted based on the following factors: *Effort—*the relative number of locks on the left box compared to the right (−2, −1, 0, 1, 2); *Stimulus—*the type of stimulus on the left (social or non-social), each stimulus was presented on the left side on 30 trials; *zAge*—zero meaned age as above; *zAge^2^*; and gender. As the analysis included all the 30 trials when the object was on the left and all the other 30 trials when social stimulus was on left, it includes a total of 60 choice trials for each participant. This was a mixed-level model where trial factors (Effort & Stimulus) are modelled as well as participant factors (ID, zAge, zAge2, gender) in a single model. We used a logistic link function to take account of the binary nature of the choices made. We tested for main effects of all the predictors and also for interactions of *Effort-by-Stimuli*; *Effort-by-zAge*; *Effort-by-zAge^2^; Stimuli-by-zAge, Stimuli-by-zAge^2^; Effort-by-Stimuli-by-zAge, and Effort-by-Stimuli-by-zAge^2^*. Results are reported in terms of the Wald statistic.

Second, we performed a *basic preference analysis*, where we collapsed across all the different levels of effort (which were balanced over trials), and calculated the percentage of trials on which a participant chose a social movie over an object movie. This provides a simple quantification of social seeking in each participant and allows us to plot the basic preference against age for all individuals. We tested if zAge or zAge^2^ could predict this basic social preference.

## 4. Results

### 4.1. Logistic regression

We found that overall choices at the group level were significantly influenced by effort (Wald *χ*^2^ = 41.04, *p* < 0.0001) whereas stimuli and gender were not significant predictors (table 3). More importantly, we found interactions between age and other factors. The choice of items could reliably be predicted by the interaction of effort and zAge (Wald *χ*^2^ = 31.07, *p* < 0.0001), interaction of stimuli and zAge (Wald *χ*^2^ = 7.00, *p* = 0.008), and interaction of stimuli and zAge^2^ (Wald *χ*^2^ = 11.35, *p* = 0.001). The two-way interaction between age and stimuli provides clear evidence for a non-monotonic developmental change in social seeking. The three-way interactions of effort by stimuli by zAge or effort by stimuli by zAge^2^ were not significant.

**Table 3.** Logistic regression models for choice.

variable	Wald *χ*^2^	d.f.	sig.
effort	41.044	4	<0.0001
stimuli	0.518	1	0.472
gender	0.042	1	0.838
zAge	0.087	1	0.768
zAge^2^	0.513	1	0.474
effort × stimuli	7.053	4	0.133
effort × zAge	31.072	4	<0.0001
effort × zAge^2^	6.479	4	0.166
stimuli × zAge	7.000	1	0.008
stimuli × zAge^2^	11.345	1	0.001
effort × stimuli × zAge	5.740	4	0.219
effort × stimuli × zAge^2^	2.239	4	0.692

### 4.2. Basic preference analysis

**Figure 2.**
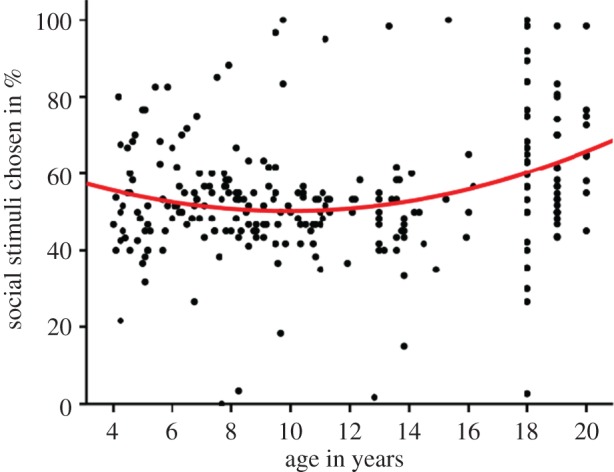
Preference for social stimuli in all participants aged 4–20 years. The red line shows the fit of the quadratic model.

An alternative visualization of the data (figure 2) shows that social preference is high in the youngest children, older adolescents and young adults in the sample, but dips around 11 years of age. We fit both linear model using Age as predictor and quadratic model using zAge and zAge^2^ as predictors. Results show that the quadratic model (*R*^2^ = 0.080, *F*_2, 252_ = 10.97, *p* < 0.0001) predicts the preference for social stimuli more reliably than the linear model (*R*^2^ = 0.045, *F*_1, 253_ = 11.79, *p* = 0.001); parameter estimates are given in table 4. We also compared the models directly (*F*_253, 252_ = 9.731, *p* = 0.002) [28] and the results confirm that the quadratic model fits the data significantly better than the linear model.

**Table 4.** Regression models for social preference and age.

	variable	*B*^a^	s.e. *B*^a^	*Β*^b^	*T*	*sig.*
quadratic	zAge	−3.020	1.204	−0.931	−2.509	0.013
	zAge^2^	0.152	0.049	1.157	3.119	0.002
linear	Age	0.685	0.199	0.211	3.434	0.001

^a^Unstandardized coefficients.

^b^Standardized coefficient.

This reinforces the primary result and shows that young children, older adolescents and young adults have a stronger preference for the social stimuli we used in this task compared to young adolescents.

### 4.3. Further analyses

We did some further analysis to see if the results from two versions of the CAM are comparable. We found that adult cohorts 1 and 4 who used CAM version 1 and 2 respectively have similar results on both logistic as well as basic choice analysis. In logistic regression, both these cohorts show significant influence of effort (cohort 1 Wald *χ*^2^ = 40.36, *p* < 0.0001 and cohort 4 Wald *χ*^2^ = 88.04, *p* < 0.0001), stimuli (cohort 1 Wald *χ*^2^ = 17.78, *p* < 0.0001 and cohort 4 Wald *χ*^2^ = 10.21, *p* < 0.001), and interaction between effort and stimuli (cohort 1 Wald *χ*^2^ = 11.23, *p* = 0.024 and cohort 4 Wald *χ*^2^ = 22.50, *p* < 0.0001). Similarly, in the basic choice analysis in which we compared the social preference (collapsed over the effort levels) for the two adult groups (cohort 1 and cohort 4), results suggest no significant difference between them (*t*_60_ = −1.619, *p* = 0.111).

We also compared the results on logistic regression with and without cohort 1 which was the only set of participants who used version 1 of the CAM task, and results are again comparable as both these groups show a significant influence of stimuli by zAge^2^ interaction on the choice behaviour (with cohort 1 Wald *χ*^2^ = 11.34, *p* = 0.001 and without cohort 1 Wald *χ*^2^ = 4.79, *p* = 0.029).

We have approximately equal numbers of male and female participants in all groups apart from cohort 3. Although the statistical analysis does not show any main effect of gender, we did additional logistic regression analyses on the male participants alone to see if there is any effect of male gender predominance in this cohort. Results show that the findings remain comparable, as we still see a significant effect of effort (Wald *χ*^2^ = 28.29, *p* < 0.0001), interaction of effort and stimuli (Wald *χ*^2^ = 12.38, *p* = 0.015), interaction of effort and zAge (Wald *χ*^2^ = 19.25, *p* = 0.001), and interaction of stimuli and zAge^2^ (Wald *χ*^2^ = 7.11, *p* = 0.008). No three-way interaction is significant. Hence, the data for this study can be interpreted irrespective of the gender of the participants.

## 5. Discussion

This study describes the developmental trajectory of social seeking from 4–20 years of age, measured using the CAM task. We found that young children, older adolescents and young adults preferred movies of people rather than objects but this social preference was not present in the young adolescents. At the group level, participants were influenced by the effort required on trials, and the preference for low effort increased with age. We consider here what these results mean for understanding typical and atypical social functioning and why it is important to have an objective tool to measure social seeking.

We used logistic regression using age as a predictor of social seeking behaviour and basic social preference exploring relationship between overall social seeking (collapsed over the effort levels) and age. Both these analyses confirm that the present data demonstrate a surprising reduction in the seeking to view social stimuli in typical young adolescents (9–12 years). There are two possible explanations for this result. These findings could be explained by a genuine, global change in the motivation to interact with others as children develop, or by a narrowing of social interest in early adolescence.

The explanation of a global change in social seeking in young adolescents is compatible with the idea of social hyper-sensitivity during this period [29]. It is known that adolescence is characterized by the experience of an imaginary audience which can result in high self-awareness and can have direct influence on social behaviours [30]. Therefore, it is possible that adolescents might undergo a change in their overall social seeking behaviour. The higher sensitivity towards social rejection in adolescents between ages 11–15 results in experience of higher distress than adults [31]. This can perhaps result in more careful social seeking behaviour than adults. Similarly, Gunther *et al.* [32] looked at the developmental trajectory of social evaluation and found that 16–25 year olds expect significantly more positive social feedback than 8–15 year olds. In another study, adolescents (13–17 years) show much higher performance interference and stress reaction to peer rejection compared to children (7–12 years), suggesting heightened social sensitivity [33]. A cross-sectional study of a large (*n* = 260) sample of typical 9–17 year olds suggested that around age 12 they report a higher desire to avoid social contacts than the younger or older age groups [34]. Finally, a study evaluating 8–22 year olds on behavioural, autonomic arousal and neurobiological activation in response to being observed by peers suggests that adolescents showed a distinct hyper-sensitivity to social evaluation compared with children and adults [35]. This resulted in a significantly high social embarrassment rating which potentially could lead to low social seeking.

The studies reviewed above suggest that most of the adolescents undergo a phase of increased self-sensitivity in response to social situations, and this could provide an explanation for our findings. However, there are limitations to this explanation. It is not yet clear if changes in self-sensitivity lead directly to changes in social seeking. Furthermore, many of these studies including ours take age as a standard milestone for pubertal changes (Sebastian *et al*. [31] is a notable exception). However, it is known that there can be a significant variation within the normal population in the age of pubertal onset [36], and changes in self-sensitivity might be more closely linked to pubertal onset than to age.

An alternative explanation for the observed dip in social seeking can also be given. The dip might reflect a narrowing of social interest rather than a global change in self-sensitivity. The stimuli in our social movies depicted smiling adults mostly aged in their 20s. Our data show that young children and young adults find these movies engaging, but the young adolescents do not. Perhaps young adolescents have a narrower social preference for their own age group (rather than all adults) so they show a reduced motivation towards viewing adults. This is consistent with previous studies showing that when assessing the risk involved in everyday situations, young adolescents may focus more on the opinion of their peer group than the opinion of adults [37]; also young adolescents make more errors when they are being observed by their peer group than by an adult experimenter [38].

Our data also relate to previous studies showing non-monotonic changes in face recognition [1] and emotion recognition [4,5] in adolescents, with an own-age bias in young adolescents [39,40] but not 3 year olds [41]. This implies that experience with a particular age-group sharpens face processing skills for that age group. Our data further imply that motivational changes driving young adolescents to associate with and attend to their peers could be an important factor influencing their social functioning. To test this, future studies could use a wider age range of actors in different movies for the social stimuli, allowing us to compare the preference for viewing same-age actors to older or younger actors. If the dip in social motivation, as observed in the present study, is a result of specific social preference for peers in young adolescents, then the age matched social stimuli might motivate them to seek social stimuli more.

### 5.1. Clinical relevance

As has been demonstrated in the current experiment, typical development may not always follow a linear progression. This means social motivation needs to be evaluated in a wide age range of typical as well as atypical participants. In most clinical settings disruption in social functioning is evaluated by comparing the reports of social functioning of patients with ‘typical social behaviour’. As parental or informant reports are known to have several inconsistencies and biases [42,43], there is a need to have a tool that can objectively evaluate current social motivation in both typical as well as atypical groups. We believe that the CAM task will be a valuable tool to quantify group/individual differences in the social seeking aspect of social motivation and track responses to treatments targeting social difficulties. It is rare to have a task that can be applied in the same way across a wide range of ages and abilities, but CAM fulfils this criterion. In the future, it will be important to see how tests of social seeking like CAM relate to real-world behaviour of seeking social contact and if CAM can be used in clinical populations to explore the developmental trajectory of social seeking.

## 6. Limitations

In this study, there were slight changes in the testing environment for different cohorts. The data were collected from public engagement events, schools and laboratory settings. In all three contexts, the participant and experimenter were seated in a quiet space without distraction from passers-by. For the younger children, the experimenter sat close to the child to ensure he/she stayed on task and finished the study, whereas less supervision was required for older participants. This might introduce some confounding variable, but it is hard to quantify and control through statistical analysis. A final limitation is that we did not screen the adult participants from cohort 1 and 4 for any current or past mental health conditions other than autistic traits. Future studies could be improved if all participants are tested in identical conditions and are screened for any mental health conditions that can have influence on the social seeking behaviour.

## 7. Conclusion

The present paper shows that changes in the motivation to seek social stimuli can be measured across the 4–20 years age range, and that social seeking might dip in young adolescents around 9–12 years. These data demonstrate the importance of measuring and understanding changes in motivation and social behaviour across a wide developmental range, in order to improve our understanding of both typical and atypical social development.

## Data accessibility

Social preference data for all participants are available at Dryad: http://dx.doi.org/10.5061/dryad.7p51p [44].
